# Replication-incompetent rabies virus vector harboring glycoprotein gene of lymphocytic choriomeningitis virus (LCMV) protects mice from LCMV challenge

**DOI:** 10.1371/journal.pntd.0006398

**Published:** 2018-04-16

**Authors:** Mutsuyo Takayama-Ito, Chang-Kweng Lim, Yukie Yamaguchi, Guillermo Posadas-Herrera, Hirofumi Kato, Itoe Iizuka, Md. Taimur Islam, Kinjiro Morimoto, Masayuki Saijo

**Affiliations:** 1 Department of virology I, National Institute of Infectious Diseases, Toyama, Shinjuku-ku, Tokyo, Japan; 2 Division of Global Infectious Diseases, Department of Infection and Epidemiology, Graduate School of Medicine, Tohoku University, Sendai, Miyagi, Japan; 3 Laboratory of Virology and Viral Infections, Faculty of Veterinary Medicine, Nippon Veterinary and Life Science University, Kyonancho, Musashino-shi, Tokyo, Japan; 4 Faculty of Pharmacy, Yasuda Women's University, Yasuhigashi, Asaminami, Hiroshima, Japan; Wistar Institute, UNITED STATES

## Abstract

**Background:**

Lymphocytic choriomeningitis virus (LCMV) causes a variety of diseases, including asymptomatic infections, meningitis, and congenital infections in the fetus of infected mother. The development of a safe and effective vaccine against LCMV is imperative. This study aims to develop a new candidate vaccine against LCMV using a recombinant replication-incompetent rabies virus (RV) vector.

**Methodology/Principal findings:**

In this study, we have generated a recombinant deficient RV expressing the LCMV glycoprotein precursor (GPC) (RVΔP-LCMV/GPC) which is lacking the RV-P gene. RVΔP-LCMV/GPC is able to propagate only in cells expressing the RV-P protein. In contrast, the LCMV-GPC can be expressed in general cells, which do not express RV-P protein. The ability of RVΔP-LCMV/GPC to protect mice from LCMV infection and induce cellular immunity was assessed. Mice inoculated intraperitoneally with RVΔP-LCMV/GPC showed higher survival rates (88.2%) than those inoculated with the parental recombinant RV-P gene-deficient RV (RVΔP) (7.7%) following a LCMV challenge. Neutralizing antibody (NAb) against LCMV was not induced, even in the sera of surviving mice. CD8+ T-cell depletion significantly reduced the survival rates of RVΔP-LCMV/GPC-inoculated mice after the LCMV challenge. These results suggest that CD8+ T cells play a major role in the observed protection against LCMV. In contrast, NAbs against RV were strongly induced in sera of mice inoculated with either RVΔP-LCMV/GPC or RVΔP. In safety tests, suckling mice inoculated intracerebrally with RVΔP-LCMV/GPC showed no symptoms.

**Conclusions/Significance:**

These results show RVΔP-LCMV/GPC might be a promising candidate vaccine with dual efficacy, protecting against both RV and LCMV.

## Author summary

Lymphocytic choriomeningitis virus (LCMV) causes infections that are often asymptomatic but can be fatal in immunocompromized persons. In addition, LCMV infection during pregnancy can cause spontaneous abortion or severe birth defects. Humans are exposed to LCMV by direct or indirect contact with wild or pet rodents such as mice, hamsters, and guinea pigs. There is no vaccine against LCMV infection. Because of the importance of cellular immunity, inactivated vaccines are not considered effective for protection against LCMV infection. In contrast, protection against rabies, one of the most lethal zoonotic diseases, depends primarily on humoral immunity. In this study, we have developed a recombinant rabies virus (RVΔP-LCMV/GPC) that cannot multiply but expresses LCMV and rabies antigens in the inoculated mice. Hence, we expected that both humoral and cellular immunity would be induced. Most of the mice (88.2%) inoculated with RVΔP-LCMV/GPC survived after a LCMV challenge, whereas only 7.7% of the empty vector inoculated mice survived. Simultaneously, RVΔP-LCMV/GPC induced strong humoral immunity against rabies virus. In conclusion, this study indicates that RVΔP-LCMV/GPC may be useful as a bivalent vaccine against LCMV and RV.

## Introduction

Arenaviruses (Genus Arenavirus, Family Arenaviridae) are enveloped, ambisense RNA viruses containing small (S) and large (L) RNA segments [1]. The S-segment encodes a nucleoprotein (NP) and a glycoprotein precursor (GPC). The L-segment encodes an RNA-dependent RNA polymerase and a small RING finger protein (Z) that functions as a matrix protein. The GPC is cleaved into 2 subunits, GP1 and GP2, and forms a mature complex [2]. Arenaviruses are divided into 2 groups, New World and Old World arenaviruses. Junin virus (New World arenavirus), Lassa virus and Lujo virus (Old World arenavirus) causes viral hemorrhagic fever (VHF) in humans, with a relatively high fatality rate [3]. Lymphocytic choriomeningitis virus (LCMV) belongs to Old World arenaviruses and causes mild and self-limited disease in humans, with symptoms such as headache, fever, chills, and muscle aches. Humans can be infected with LCMV through exposure to rodent feces. LCMV also can be transmitted via solid organ transplantation and causes fatal infections in immunosuppressed recipients [4,5]. In addition, LCMV infection during pregnancy can result in abortion and cause congenital defects in infants infected in utero [6,7]. Therefore, a vaccine to protect humans against arenavirus-associated VHF and LCMV infection is needed. An inactivated, whole-virion vaccine is reported to strongly induce a humoral immune response against viral antigens but fails to protect animals from a lethal challenge of Lassa [8] or Junin [9] virus. DNA or live-attenuated vaccines expressing the arenavirus GPC and/or NP would be appropriate vaccine candidates for eliciting effective cellular immunity against the arenavirus infection. To date, only the live-attenuated Junin virus vaccine has been developed; this vaccine is presently used in Argentina [10]. No vaccines for other arenaviruses have been approved in clinical use, although candidate vaccines against Lassa virus infection has been reported. Recombinant vaccinia viruses [11,12,13,14], recombinant vesicular stomatitis viruses (VSV) [15], virus-like particles [16,17], and DNA vaccines [18] have been shown to provide complete or partial protection against lethal Lassa virus challenge. In the quest for an LCMV vaccine, recombinant viral vectors [19], DNA vaccines [20,21], virus-like particles [22], and an attenuated live vaccine [23] have been developed. These vaccines for arenaviruses target the NP and GPC proteins as antigens, and studies using recombinant virus in a nonhuman primate model suggest that the full-length GPC is necessary and sufficient for protection [14,15,24].

Rabies virus (RV) causes rabies, a zoonotic viral disease of the central nervous system that can infect almost every mammalian species. Once symptoms appear, the mortality rate is 100%. It is estimated that approximately 55,000 individuals die of rabies each year [25]. Rabies vaccine can prevent rabies at a rate of nearly 100%, if it is administered to a person immediately after they have been exposed. Inactivated vaccines against rabies are widely used globally. These vaccines were developed from laboratory strains, which efficiently propagated in cultured cells and were highly attenuated by serial passage in animal brains, chicken embryo, and cultured cells. RV belongs to the genus Lyssavirus of the family Rhabdoviridae and has unsegmented, negative-sense RNA as its genome. Reading from the 3′ to 5′ end, the genome encodes the genes of five structural proteins: nucleoprotein (N), phosphoprotein (P), matrix protein (M), glycoprotein (G), and polymerase (L) [26]. The genomic RNA plus the N, P, and L proteins form the ribonucleoprotein (RNP), which is the component that is active in transcription and replication. The M protein and the G protein are membrane-associated proteins. A reverse genetics system for rabies was developed by Schunel et al. in 1994 [27]. This system has been extensively used not only as a tool for investigating the virulence of RV but also as a viral vector for other antigens and a tracer for studying neuronal networks [28,29]. Recombinant RVs, which lack one of the viral genes, were shown to be safe and immunogenic in mice and nonhuman primates [29]. In fact, replication-incompetent RV was shown to be a powerful tool for developing RV vaccines [30,31,32,33]. Additionally, M- or G-deficient RVs with the antigens of foreign pathogens have been used as vectors for vaccines against simian immunodeficiency virus [34] and Ebola virus, respectively [35,36]. Moreover, a previously developed P-gene-deficient rabies virus (RV) (RVΔP) was confirmed to be efficacious in protecting mice against lethal RV infection [37,38]. We hypothesized that RVΔP, which neither replicates nor produces infectious RV, might be suitable as a vector for the development of recombinant RVΔP that expresses a foreign gene.

HEP-Flury is the parental strain of RVΔP (GenBank: AB085828.1); it has been applied for human use in an inactivated rabies vaccine as it is one of the most attenuated RV strains. HEP-Flury causes no symptoms in adult mice, but kills suckling mice infected by intracerebral inoculation [39]. In contrast, RVΔP does not kill suckling mice, indicating a marked further attenuation in its *in vivo* pathogenicity. Despite such high attenuation, the inoculation of mice with RVΔP induces a high level of NAb and confers protective immunity against lethal RV infection [37]. In addition, Cenna et al. reported that P-gene-deficient RV elicited a rapid and potent IgG2a-dominated immune response and was completely safe in Rag2 knock-out mice inoculated intramuscularly [40]. It has been reported that an RVΔM vector expressing RV-G protein more efficiently elicited protective immunity against RV than RVΔP and that RVΔM is effective as an RV vaccine even upon inoculation at a low dose, namely, 10^3^ focus-forming units (FFU)/mouse. Nonetheless, RVΔP shows the same efficacy as RVΔM at a high dose, namely, 10^5^ FFU/mouse [31]. The main advantage of RVΔP compared with RVΔM is the ease of establishing and maintaining stable RV-P protein expression in cells. Since RV-M protein is known to be highly cytotoxic [41], it would be necessary to use an expression control system to establish cell lines that stably express it. In contrast, RV-P protein-expressing cells are easy to establish without the need for any complicated system [38]. In addition, since RV-P protein was identified as a major and multifunctional type 1 interferons (IFNs) antagonist [42], the RVΔP vector probably cannot inhibit type 1 IFNs expression in infected cells. Although this would probably be unfavorable for viral replication, it could be suitable for a vaccine, since it has been reported that CD8^+^ T cells were significantly activated in mice immunized with an RV vaccine vector expressing IFN-β as an adjuvant [43]. Since type 1 IFNs directly promotes the proliferation of antigen-specific CD8^+^ T cells [44], it is expected that RVΔP would stimulate type 1 IFN expression and enhance host immune responses.

In this study, we have developed replication-incompetent RVΔP-LCMV/GPC that contains the LCMV-GPC gene in a P-gene-deficient RV genome. The efficacy of RVΔP-LCMV/GPC in protecting mice from LCMV infection was evaluated. Anti-RV NAb titers were measured. Furthermore, the safety of RVΔP-LCMV/GPC was evaluated by suckling mice inoculated intracerebrally with RVΔP-LCMV/GPC. The mechanism of vaccine protection using a recombinant RVΔP expressing LCMV antigen was evaluated virologically and immunologically.

## Materials and methods

### Cell cultures

This study used Neuro-2a cells gifted from Dr. Satoshi Inoue [National Institute of Infectious Diseases (NIID), Tokyo, Japan] and HEK-293 cells purchased from RIKEN BioResource Center (Ibaraki, Japan), and Vero cells (ATCC CCL-81) purchased from ATCC (Manassas, VA, USA). Neuro-2a cells were grown in Dulbecco’s modified Eagle’s medium (D5796, D-MEM, Sigma-Aldrich, St. Louis, MO, USA) supplemented with 10% fetal bovine serum (FBS; Biowest, Nuaillé, France), 100 U/mL penicillin, and 100 μg/mL streptomycin (Thermo Fisher Scientific Inc. Kanagawa, Japan) (D-MEM-10FBS). HEK-293 cells and Vero cells were grown in Eagle’s minimum essential medium (M4655, E-MEM, Sigma-Aldrich) containing 10% (E-MEM-10FBS) or 5% (E-MEM-5FBS) FBS, 0.1-mM nonessential amino acids, 100 U/mL penicillin, and 100 μg/mL streptomycin (Thermo Fisher Scientific Inc.). BHK-21 cell lines expressing RV P protein (BHK-P) [37] were grown in D-MEM-10FBS and 200 μg/mL Zeocine. During exposure to RV, BHK-P cells were cultured in D-MEM containing 2% FBS and antibiotics (D-MEM-2FBS). The recombinant RVΔP-LCMV/GPC developed in this study was also grown in BHK-P cells. Neuro-2a and BHK-P cells were passaged generally twice per week at split ratios of 1:6 and 1:20, respectively. HEK-293 and Vero cells were passaged once per week with a split ratio of 1:8 and 1:10, respectively. Neuro-2a cells were seeded into 24- or 96-well culture plates (Techno Plastic Products AG, Trasadingen, Switzerland) at a density of 6 × 10^4^ cells/cm^2^ 1 day before virus inoculation. BHK-21, BHK-P, and HEK-293 cells were seeded into plates at a density of 1 × 10^5^ cells/cm^2^. All cultures were cultured in a humidified incubator at 37°C with 5.0% CO_2_.

### Generation of RVΔP with expression of the LCMV-GPC

The full-length GPC gene of the LCMV WE strain (LCMV-WE) was amplified and inserted into the plasmid p5.1-defP, which has BsiWI and PstI restriction enzyme sites on the N-P intergenic region of the p3.1-defP [37]. The plasmid was designated as p5.1-defP-LCMV/GPC. Recombinant virus RVΔP-LCMV/GPC was rescued with p5.1-defP-LCMV/GPC and 4 helper plasmids (pH-N, pH-P, pH-G, and pH-L) using a previously described reverse genetics method [45].

### Generation of adenovirus expressing the LCMV-GPC

To generate the recombinant adenovirus expressing the GPC of the LCMV-WE, the GPC gene was amplified and cloned into SwaI digested cosmid vector pAxCAwtit2 (WAKO, Osaka, Japan) and named pAx-LCMV/GPC. Recombinant adenoviruses, Ax-LCMV/GPC and Ax-empty, were generated as described before [46]. Briefly, HEK-293 cells were transfected with linearized cosmid vectors pAx-LCMV/GPC or pAxCAwtit2 (RDB05213, RIKEN BioResource Center), respectably, and cloned by limiting dilution.

### Viruses

**Fig 1:**
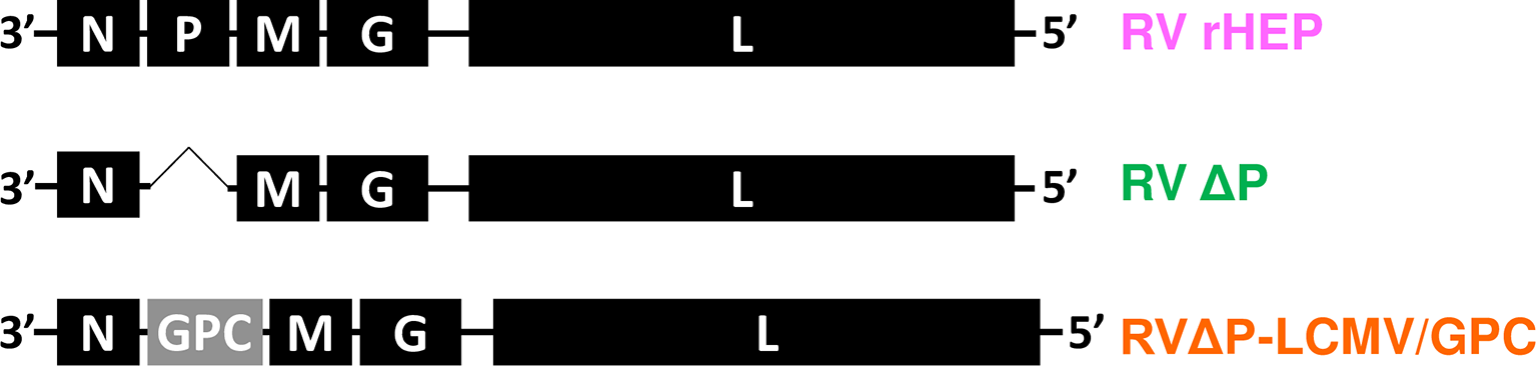
Schematic diagrams of the recombinant RVs genome constructs. Recombinant HEP-Flury (rHEP) has a complete genome of RV HEP-Flury strain (upper), RVΔP lacks the RV-P gene (middle). RVΔP-LCMV/GPC harbors the LCMV-GPC gene after the RV-N gene of the genome.

A wild type recombinant HEP-Flury strain (rHEP) was generated from full-length cDNA of the HEP-Flury strain by reverse genetics method as described previously [45] (Fig 1). The HEP-Flury strain is highly attenuated and is used as inactivated rabies vaccine for humans in Japan. RVΔP, which lacks the P gene in the RV genome [37], and newly prepared recombinant RVΔP-LCMV/GPC were also generated by reverse genetics method in BHK-P cells. After viral inoculation, inoculated cells were cultured in a humidified incubator at 33°C with 5.0% CO_2_ because RV grows better at temperatures below 37°C [47]. The RVΔP and RVΔP-LCMV/GPC used in the animal experiments were propagated in hyper flasks (Corning, NY, USA), concentrated by precipitation with 7% polyethylene glycol 6000 (PEG: WAKO, Osaka, Japan), purified by ultracentrifugation (83,000×g, 90 min) with 60% and 20% sucrose (WAKO), and treated with Amicon Ultra-15 (Merck Corporation, Darmstadt, Germany). The purified virus stocks were stored at −80°C until use. To inactivate RVΔP-LCMV/GPC, the purified virus stock was irradiated with UV light in a Falcon 35-mm culture dish (Corning) for 15 min. Virus inactivation was confirmed by absence of positive fluorescent focus by focus-forming assay using inoculated BHK-P cells.

LCMV-WE was grown in BHK and Vero cells as described previously [48]. Briefly, cells were seeded in tissue culture bottles and infected with each of the recombinant viruses at a multiplicity of infection (MOI) of 0.03 and incubated for 3 days. After incubation, the culture medium was collected and centrifuged; the supernatant fraction stored at −80°C until use. Recombinant adenoviruses were grown in HEK-293 cells and titrated as described elsewhere [49]. Recombinant adenoviruses were enriched and purified using ViraBind Adenovirus purification kit (Cell Biolabs Inc., San Diego, CA, USA). The titer of Ad was evaluated by standard plaque forming assay with HEK-293 cells [50].

### Mice

Specific pathogen-free 3-week-old female inbred C57BL/6j mice were purchased from Japan SLC Inc. (Shizuoka, Japan) and allowed to acclimate for 1 week. One-day-old specific pathogen-free outbred ICR mice were purchased from Japan SLC Inc. Eight suckling mice were placed in a cage with their untreated mother.

### Immunofluorescent assay (IFA)

BHK-P cells seeded on culture plates were inoculated with each of the viruses at 37°C for 1 h and cultured in D-MEM-2FBS at 33°C for 48 h. After incubation, the cells were fixed with Mildform10N (WAKO, Osaka, Japan) for 20 min and washed with phosphate buffered saline solution (PBS) 5 times. The cells were permeabilized in 0.5% Triton-X100 for 20 min at room temperature and washed with PBS 3 times. To detect the LCMV-GPC expression, cells blocked in PBS containing 2% FBS for 1 h were stained for 1 h at 37°C with an anti-LCMV-GP1 mAb (clone KL25) [51] (kindly provided by Dr. Daniel Pinschewer, University of Basel), washed 3 times with PBS, and stained with the secondary antibody Dylight 549-conjugated polyclonal anti-mouse IgG(H+L) (Vector Laboratories, CA, USA). The cells were observed with a fluorescence microscope OLYMPUS X-81 (Olympus Co., Tokyo, Japan). Images were captured by an ORCA-R2 (Hamamatsu Photonics K.K. Shizuoka, Japan) and colored with LuminaVision (MITANI Corporation, Tokyo, Japan). For detection of RV-N, the PBS-washed cells were stained with fluorescein isothiocyanate (FITC)-labeled anti-RV mAb (Fujirebio Inc, Tokyo, Japan) for 1 h at 37°C. The cells were also observed as described above.

### Virus titration

Titers of RVΔP and newly generated RVΔP-LCMV/GPC were determined by focus assay in BHK-P cells. BHK-P cells cultured in 96-well plates for 1 day were inoculated with each virus solution diluted 10-fold serially and incubated for 3 days at 33°C. The cells were then fixed with 80% acetone for 20 min at room temperature. Fixed cells were stained with FITC-labeled anti-RV mAb, observed under a fluorescence microscope, and foci were counted. The infectious doses of RVΔP and RVΔP-LCMV/GPC were calculated and shown as FFU per mL.

The infectious titers of LCMV solution were determined using the plaque assay as described elsewhere [48]. Briefly, Vero cells were seeded onto 6-well plates and were inoculated with 10-fold serially diluted virus solutions. After removing the virus solutions, 0.5% agarose overlay medium were added on the cells. The cells were incubated for 4 days at 37°C and stained with neutral red. The infectious dose was calculated and shown as plaque forming unit (PFU) per mL. Besides plaque assay, the infectious titers of LCMV were determined by focus forming assay described before [52] for neutralizing antibody assay. Briefly, Vero cells seeded in 96-well plates were inoculated with each of the virus solutions 10-fold serially diluted and then incubated for 40–48 h at 37°C. The cells were fixed with Mildform10N and permeabilized with 0.5% Triton-X100 and were stained with mAb KL-25, and then HRP-anti-mouse IgG (H+L) (Thermo Fisher Scientific Inc.) after washing the cells. Foci in the cells were counted. The infectious dose was calculated and shown as FFU per mL.

### Western blotting

Cells infected with either of RVΔP-LCMV/GPC or RVΔP were incubated for 48 h at 33°C. Cells were then washed with PBS twice and lysed with lysis buffer and centrifuged at 14,000×g for 10 min at 4°C. Sample supernatants were mixed with an equal volume of sample buffer containing 2-ME and incubated at 98°C for 2 min. Samples were separated with precast 10% gel SDS-PAGE (ATTO, Tokyo, Japan) and transferred onto polyvinylidene difluoride (PVDF) membranes. Membranes were incubated with anti-RV-G mAb15-13 [53], kindly distributed by Dr. Nobuyuki Minamoto (University of Gifu), or the anti-LCMV-GP1 mAb. After incubation followed by washing in PBS, the membranes were incubated with HRP anti-mouse IgG (H+L) (Thermo Fisher Scientific Inc.), stained with SuperSignal West Femto (Thermo Fisher Scientific Inc.), and visualized with LAS-3000 (Fujifilm, Tokyo, Japan).

### Evaluation of RVΔP-LCMV/GPC in the protection of mice after intracerebral LCMV infection

Groups of five mice were intraperitoneally inoculated with 10^6^ FFU/0.1 mL of RVΔP, RVΔP-LCMV/GPC, UV-irradiated RVΔP-LCMV/GPC, or PBS containing 2% BSA twice at 1-week intervals. Mice were infected with 10 PFU of LCMV-WE under isoflurane anesthesia 1 week after the last inoculation. After challenge, the mice were observed for 2 weeks. The number of surviving mice was recorded daily. To determine the serum anti-LCMV and anti-RV NAb titers, blood samples were collected from the facial vein of the mice using an animal lancet (Medipoint Inc, Mineola, NY, USA) 1 day before the days of inoculation, challenge, and completion of the observation period.

### Safety profile test

Groups of eight suckling mice were inoculated intracerebrally with 30 μL of rHEP RV, RVΔP, or RVΔP-LCMV/GPC, each of which contained 2 × 10^5^ FFU of the viruses, using a double-hub needle and 0.3 mL glass syringe (Hoshiseido Medical Appliance Industry Co., Ltd., Tokyo, Japan). The suckling mice were observed for clinical signs for 3 weeks.

### Anti-LCMV NAb assay

The infectious dose of LCMV was determined using a focus reduction assay [52]. Sera were diluted 1:16 with E-MEM-5FBS and heat-inactivated at 56°C for 30 min and were further serially 2-fold diluted in E-MEM-5FBS. Each of the diluted serum samples was mixed with an equal volume (0.05 mL) of virus solution containing 50 FFU of LCMV-WE in a well of a 96-well plate. After incubation at 37°C for 1 h, 2.5 ×10^5^ Vero cells in 0.05 mL were added to each well and incubated for 4–6 h. Then, 0.1 mL of overlay medium (E-MEM-5FBS with 1% methylcellulose) was added to each well. The cells were further incubated for 42–48 h at 37°C. The cells were fixed with 4% formaldehyde and permeabilized with 0.5% Triton X-100. FBS (10%) was added to each well and incubated for 60–90 min at 37°C for blocking. After washing with PBS, the cells were stained with the anti-LCMV-GP1 mAb, followed by treatment with HRP-labeled anti-mouse IgG (H+L) (Thermo Fisher Scientific Inc.). DAB solution (Wako Pure Chemical Industries, Ltd. Osaka, Japan) with 0.1M imidazole (Wako Pure Chemical Industries) was added and incubated at room temperature for 10–30 min. Neutralization was determined by counting the number of foci in each well. The NAb titer was defined as the reciprocal of the highest dilution level at which the number of foci was less than half that of the control (wells infected with a mixture of LCMV and pre-immunized serum collected from the same mice).

### IFA for detecting anti-LCMV IgG

The IgG antibody titers of LCMV in mouse sera were determined using an IFA. HEK-293 cells were inoculated with Ax-LCMV/GPC or Ax-empty and incubated at 37°C for 48 h. The cells were collected, mixed with two volumes of mock-inoculated HEK-293 cells, and placed onto 48-well slide glasses. Cells were fixed with 4% formaldehyde, permeabilized with 0.5% Triton X-100, and stored at −20°C until use. As a negative control, HEK-293 cells infected with empty adenovirus were used. Sera were serially diluted 2-fold (from 1:8 to 1:2048) and used as the primary antibody, and 400-fold diluted FITC-labeled anti-mouse IgG(H+L) (Cappel, MP Biochemicals LLC, Santa Ana, CA, USA) was used as the secondary antibody. The antibody titers against the LCMV-GPC were determined in terms of the highest dilution of serum for which IFA positivity of cells could be demonstrated.

### Anti-RV NAb assay

Titers of anti-RV NAb in mouse sera were determined using a modified rapid fluorescent focus-forming inhibition test. Briefly, sera were diluted 16-fold with D-MEM-10FBS and inactivated at 56°C for 30 min. The heat-inactivated sera were serially diluted 2-fold and mixed with an equal volume (0.05 mL) of virus solution containing 50 FFD_50_ (50% focus-forming dose). After incubation at 37°C for 1 h, 2 ×10^5^ cells/0.05 mL of Neuro-2a cells were added. Cells were incubated at 37°C for 48 h and then fixed with 80% acetone for 20 min at room temperature. The cells were stained with FITC-labeled anti-RV antibody for 40 min at 37°C. The NAb titer was defined as the highest serum dilution that neutralizes 50% of the challenge virus. This value was normalized to international units (IU) using the World Health Organization anti-rabies immunoglobulin.

### Evaluation of RVΔP-LCMV/GPC in the protection of CD8+ T-cell-depleted mice from LCMV infection

Groups of six mice were intraperitoneally inoculated with 10^6^ FFU/ 0.1 mL of RVΔP-LCMV/GPC. The mice were inoculated again with the same amount of RVΔP-LCMV/GPC 3 weeks after the first inoculation. Four days and 7 days after the last RVΔP-LCMV/GPC inoculation, the mice were injected with 500 μg of anti-mouse CD8α Clone 53–6.72 (Bio X Cell, Lebanon, NH, USA) for CD8+ T-cell depletion or 500 μg of Rat IgG2b isotype (anti-Trinitrophenol Clone 2A3, Bio X Cell) as the control. One week after the last RVΔP-LCMV/GPC inoculation (3 days after the anti-mouse CD8α Clone 53–6.72 or 500 μg of Rat IgG2b isotype control injection), the mice were infected intracerebrally with 10 PFU of LCMV-WE and observed for 3 weeks.

### Ethics statement

The animal studies were carried out in strict accordance with the Guidelines for Proper Conduct of Animal Experiments of the Science Council of Japan and with animal husbandry and welfare regulations. All animal experiments were reviewed and approved by the Committee on Experimental Animals at the National Institute of Infectious Diseases (NIID) (approval nos. 214091, 215084, 114122, and 116130). All mice infected with LCMV-WE were handled in biosafety level 3 animal facilities, in accordance with the guidelines of the NIID. The mice were inoculated with virus under proper anesthesia with isoflurane. During the observation period, the mice were monitored daily, and moribund mice were euthanized with isoflurane.

### Statistical analysis

The data were analyzed using JMP 11 (SAS) software. The growth of virus was analyzed using the Kruskal–Wallis test. Differences in survival and recurrences between groups were compared using Kaplan–Meier curves and tested using the log-rank test. The Steel–Dwass nonparametric test was used for multiple comparisons of NAb titers.

## Results

### Construction of RVΔP-LCMV/GPC and expression of the LCMV-GPC protein in BHK-P cells infected with RVΔP-LCMV/GPC

**Fig 2:**
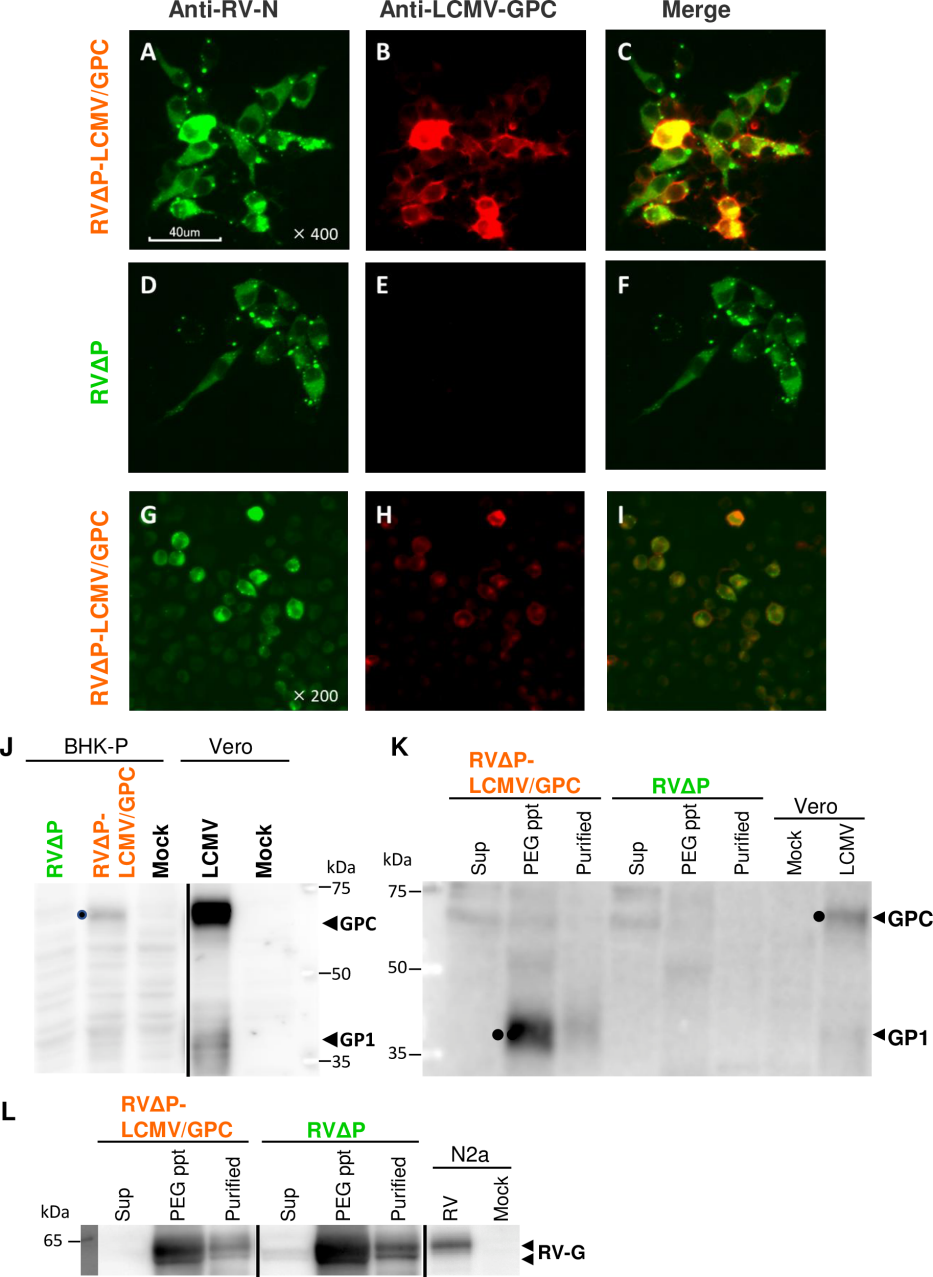
Confirmation of RV-N and LCMV-GP expression. BHK-P cells were inoculated with RVΔP-LCMV/GPC (A–C) or RVΔP (D–F) at an MOI of 0.1 and incubated at 33°C for 48 h. Cells were stained with anti-RV N mAb or the anti-LCMV-GP1 mAb. Original magnification, 400×. (G–I) RV-N and the LCMV-GPC were detected in Neuro-2a cells inoculated with RVΔP-LCMV/GPC at an MOI of 8 at 48 h post inoculation). Original magnification, 200×. (J) GPC (70 KDa) and GP1 (40 KDa) proteins were detected in BHK-P cells infected with RVΔP-LCMV/GPC by western blot using the anti-LCMV-GP1 mAb. (K–L) The cultured medium (Sup) of cells infected with RVΔP-LCMV/GPC or RVΔP were PEG precipitated (PEG ppt) and purified (Purified) by ultracentrifugation and stained with the anti LCMV-GP1 Mab (K) or the anti-RV G mAb (L). The lysates of mock-inoculated Vero cells and LCMV-WE -inoculated Vero cells were used as negative and positive controls, respectively (K). Lysates of mock-inoculated Neuro-2a cells and RV HEP-Flury strain-infected Neuro-2a cells were used as negative and positive controls, respectively (L).

RVΔP-LCMV/GPC was successfully generated (Fig 1). No mutations were found in the nucleotide sequence of the LCMV-GPC gene inserted into the virus. BHK-P cells were inoculated with RVΔP-LCMV/GPC at an MOI of 0.1 and incubated at 33°C for 48 h. LCMV-GP1-positive cells were observed in BHK-P cells infected with RVΔP-LCMV/GPC (Fig 2B) but not in those infected with RVΔP (Fig 2E). In contrast, RV-N antigen-positive foci were observed both in RVΔP-LCMV/GPC- and RVΔP-infected BHK-P cells (Fig 2A and 2D). LCMV-GP1 was also detected in RVΔP-LCMV/GPC-infected Neuro-2a cells (Fig 2H), which do not supply RV-P protein. The expression of the LCMV-GPC- and GP1 proteins in RVΔP-LCMV/GPC-infected BHK-P cells was examined by western blotting (Fig 2J). The LCMV-GPC was definitely detected in RVΔP-LCMV/GPC-infected BHK-P cells, although it was lower expression level than that in LCMV-WE-infected Vero cells. GP1 antigen was detected in the PEG-precipitated fraction and the purified virion of RVΔP-LCMV/GPC (Fig 2K). In contrast, the LCMV-GPC and GP1 were not detected in the PEG-precipitated fraction and the purified virion of RVΔP (Fig 2K). RV-G was detected in the PEG-precipitated and the purified virions of RVΔP-LCMV/GPC, RVΔP and HEP infected Neuro-2a cells (Fig 2L).

### Growth property of RVΔP-LCMV/GPC and RVΔP in BHK-P cells

**Fig 3:**
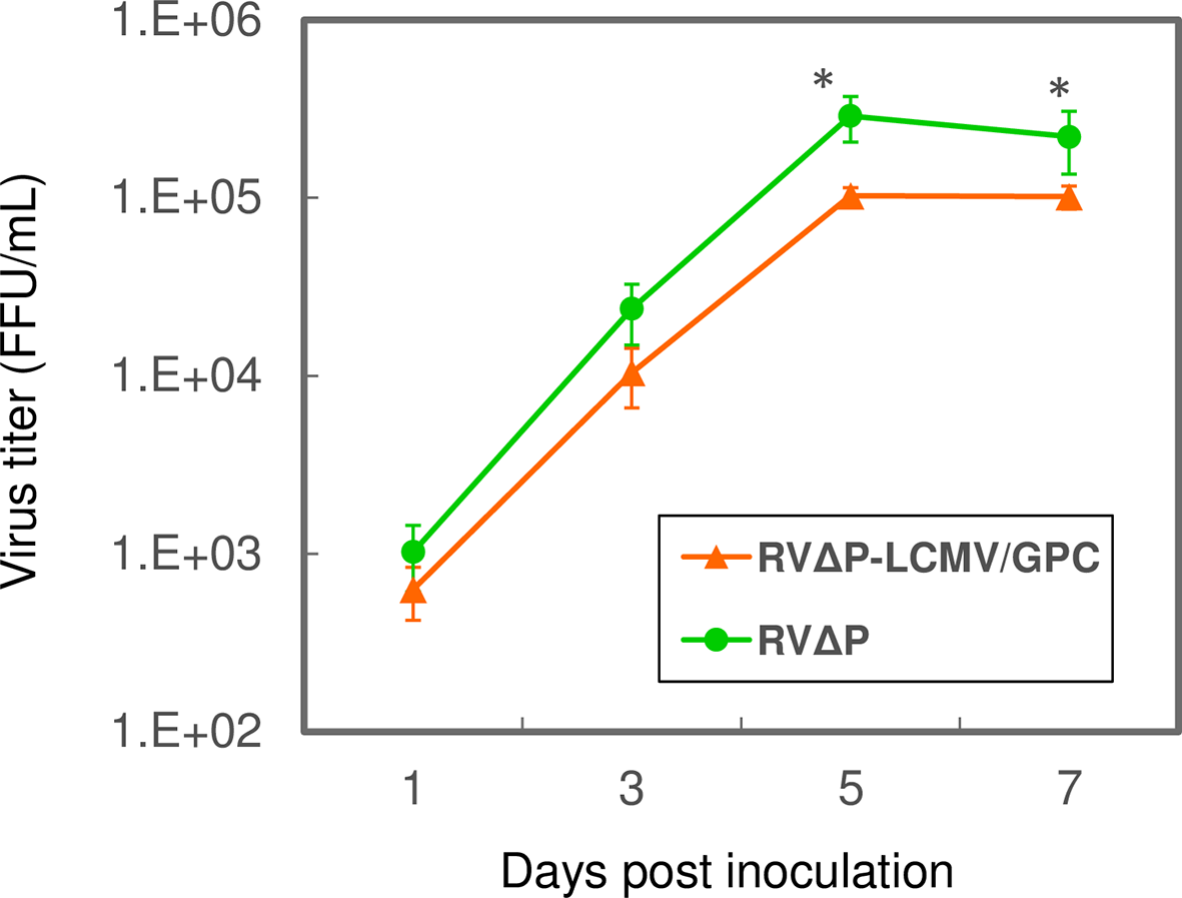
Growth kinetics of RVΔP-LCMV/GPC and RVΔP in BHK-P cells. BHK-P cells were inoculated with RVΔP-LCMV/GPC or RVΔP at an MOI of 0.01. Culture supernatants were harvested at 1, 3, 5, and 7 days post inoculation, and virus titers were determined using BHK-P cells. The data were expressed as mean ± SE of 4 independent experiments. Asterisks indicate a significant difference (p < 0.05).

BHK-P cells were inoculated with RVΔP-LCMV/GPC or RVΔP at an MOI of 0.01 and incubated at 33°C for 7 days. Titers of the progeny viruses were determined on day 1, 3, 5 and 7 days (Fig 3). These strains demonstrated similar growth curves, while the growth capacity differed significantly between RVΔP-LCMV/GPC and RVΔP strains on day 5 (p = 0.0459) and 7 (p = 0.0459), The titers of RVΔP-LCMV/GPC and RVΔP were highest on day 5, reaching a maximum titer of 10^5^ FFU/mL.

### RVΔP-LCMV/GPC showed no pathogenicity in suckling mice infected by intracerebral inoculation

**Fig 4:**
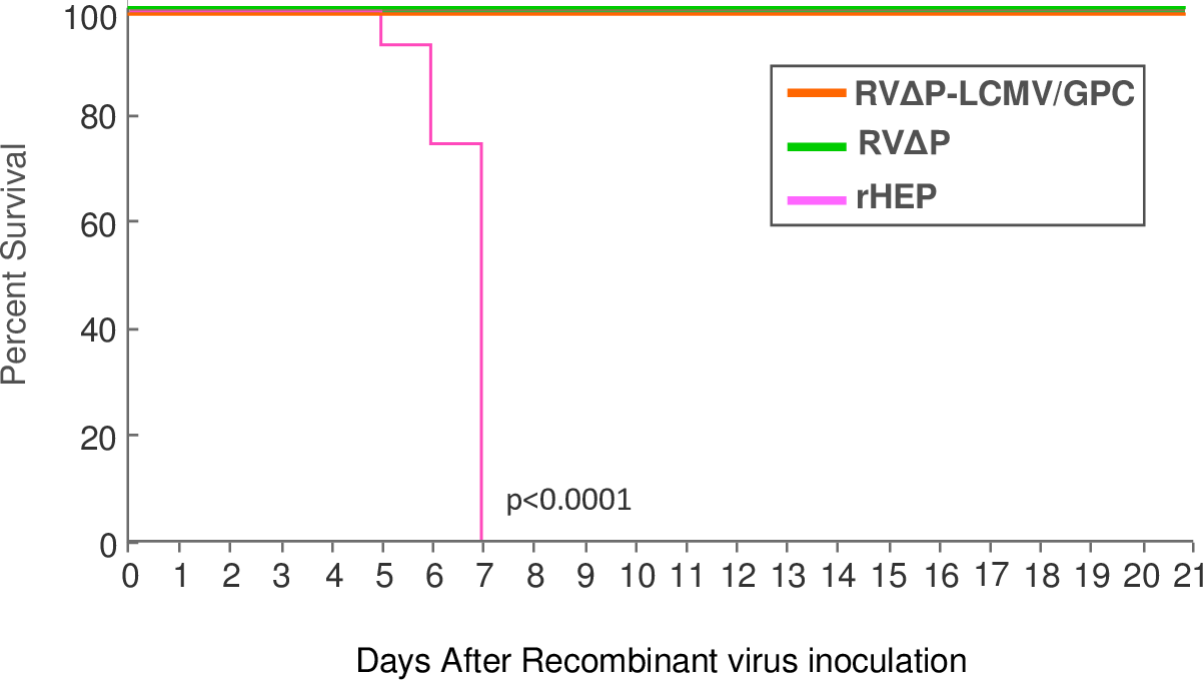
Safety profile of RVΔP-LCMV/GPC. Suckling mice (4-day old) were inoculated with RVΔP-LCMV/GPC (Orange), RVΔP (Green), or rHEP (Pink) by intracerebral inoculation and observed for 3 weeks. Euthanasia was performed when the mouse was considered to have reached a moribund stage. Number of mice in each group was 16.

To address the safety of RVΔP-LCMV/GPC, 4-day old suckling ICR mice were inoculated intracerebrally with RVΔP-LCMV/GPC, RVΔP, or rHEP and observed for 3 weeks. Mice inoculated with RVΔP-LCMV/GPC or RVΔP showed no clinical signs during the observation period (Fig 4). In contrast, all mice inoculated with rHEP died or were sacrificed moribund within 7 days of inoculation. Statistically significant differences were observed in survival rates between mice inoculated with rHEP and those inoculated with RVΔP-LCMV/GPC or RVΔP.

### Intraperitoneal inoculation with RVΔP-LCMV/GPC protected mice against LCMV challenge

**Fig 5:**
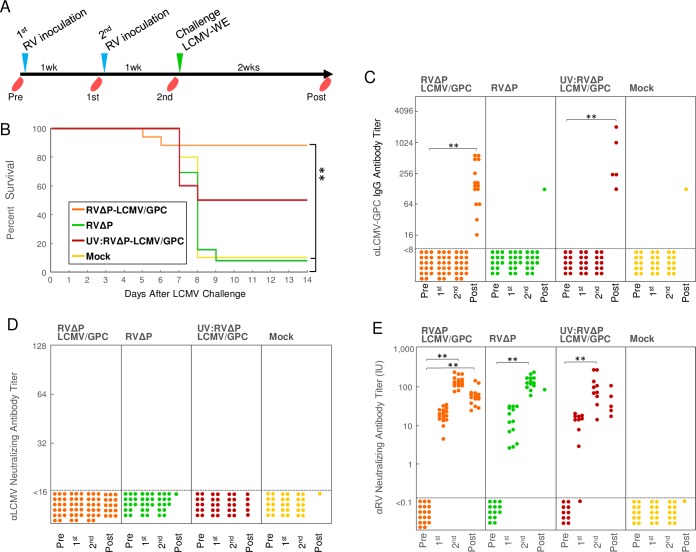
Survival curves and antibody titers in mice inoculated with RV vectors before LCMV-WE infection. (A) RV inoculation, challenge, and blood collection schedules in C57BL/6 mice. Mice were inoculated twice with RVΔP-LCMV/GPC, RVΔP, UV-irradiated RVΔP-LCMV/GPC, or PBS (mock-inoculated control) at 1-week intervals. One week after the last inoculation of RVs, mice were challenged with 10 PFU of LCMV-WE and observed for 2 weeks. Serum was collected from mice inoculated with RVs 1 day before the first RV inoculation (Pre) and second RV inoculation (1st) and at the LCMV challenge (2nd). Serum was collected from surviving mice on the last day of the 2-week observation period (Post). (B) The survival curve of mice inoculated with RVΔP-LCMV/GPC, RVΔP, UV-irradiated RVΔP-LCMV/GPC or PBS. Asterisks indicate a significant difference (p < 0.001). The titers of IgG antibody against the LCMV-GPC (C) and NAb against LCMV (D) and RV (E) in serum of mice inoculated with RVΔP-LCMV/GPC (n = 17), RVΔP (n = 13), UV-irradiated RVΔP-LCMV/GPC (n = 10), or PBS (n = 10).

To examine the efficacy of RVΔP-LCMV/GPC as a vaccine against LCMV, mice were intraperitoneally inoculated twice with RVΔP-LCMV/GPC, RVΔP, or UV-irradiated RVΔP-LCMV/GPC at 1-week intervals. One week after the last immunization, the mice were intracerebrally inoculated by injection with 10 PFU of LCMV-WE (Fig 5A). As shown in Fig 5B, the survival rate of mice inoculated with RVΔP-LCMV/GPC was 88.2% (15 out of 17 mice), while those of mice inoculated with RVΔP, UV-irradiated RVΔP-LCMV/GPC, and mock inoculated were 7.7% (1/13), 50% (5/10), and 10% (1/10), respectively. The survival rate of mice inoculated with RVΔP-LCMV/GPC was significantly higher than that of mice inoculated with RVΔP (p < 0.0001) or mock-inoculated controls (p = 0.0005). No significant difference was observed between RVΔP-LCMV/GPC and UV-irradiated RVΔP-LCMV/GPC (p = 0.0552). Inoculation with UV-irradiated RVΔP-LCMV/GPC exhibited a moderate efficacy for the LCMV challenge infection. These survival rates did not show significant difference between mice inoculated with UV-irradiated RVΔP-LCMV/GPC and those inoculated with RVΔP (p = 0.1306) or mock- inoculated controls (p = 0.3136).

### Induction of IgG antibody against the LCMV-GPC and NAbs against LCMV and RV

Anti-LCMV and anti-RV antibody titers in the sera were examined in mice inoculated with RVΔP-LCMV/GPC, RVΔP, UV-irradiated RVΔP-LCMV/GPC, or PBS. As shown in Fig 5C, IgG titers against the LCMV-GPC were undetectable in the sera of mice inoculated with RVΔP-LCMV/GPC, RVΔP, or UV-irradiated RVΔP-LCMV/GPC, even after a second inoculation. But all mice that survived after LCMV challenge have definitely elicited the antibodies against the LCMV-GPC in the sera. Titers of IgG anti-LCMV-GPC antibody were up to 1:2048 in the sera of surviving mice. However, the NAb titers against LCMV did not detect throughout the observation period any of the groups (Fig 5D). In contrast, the NAb titers against RV increased after inoculation with RVΔP-LCMV/GPC, RVΔP, or UV-irradiated RVΔP-LCMV/GPC mice and were considerably higher than those of mock-inoculated mice (Fig 5E). There were no statistically significant differences among the anti-RV NAb titers of mice inoculated with RVΔP-LCMV/GPC, RVΔP, or UV-irradiated RVΔP-LCMV/GPC.

### CD8+ T cells seem to be critical for protection in mice inoculated with RVΔP-LCMV/GPC

**Fig 6:**
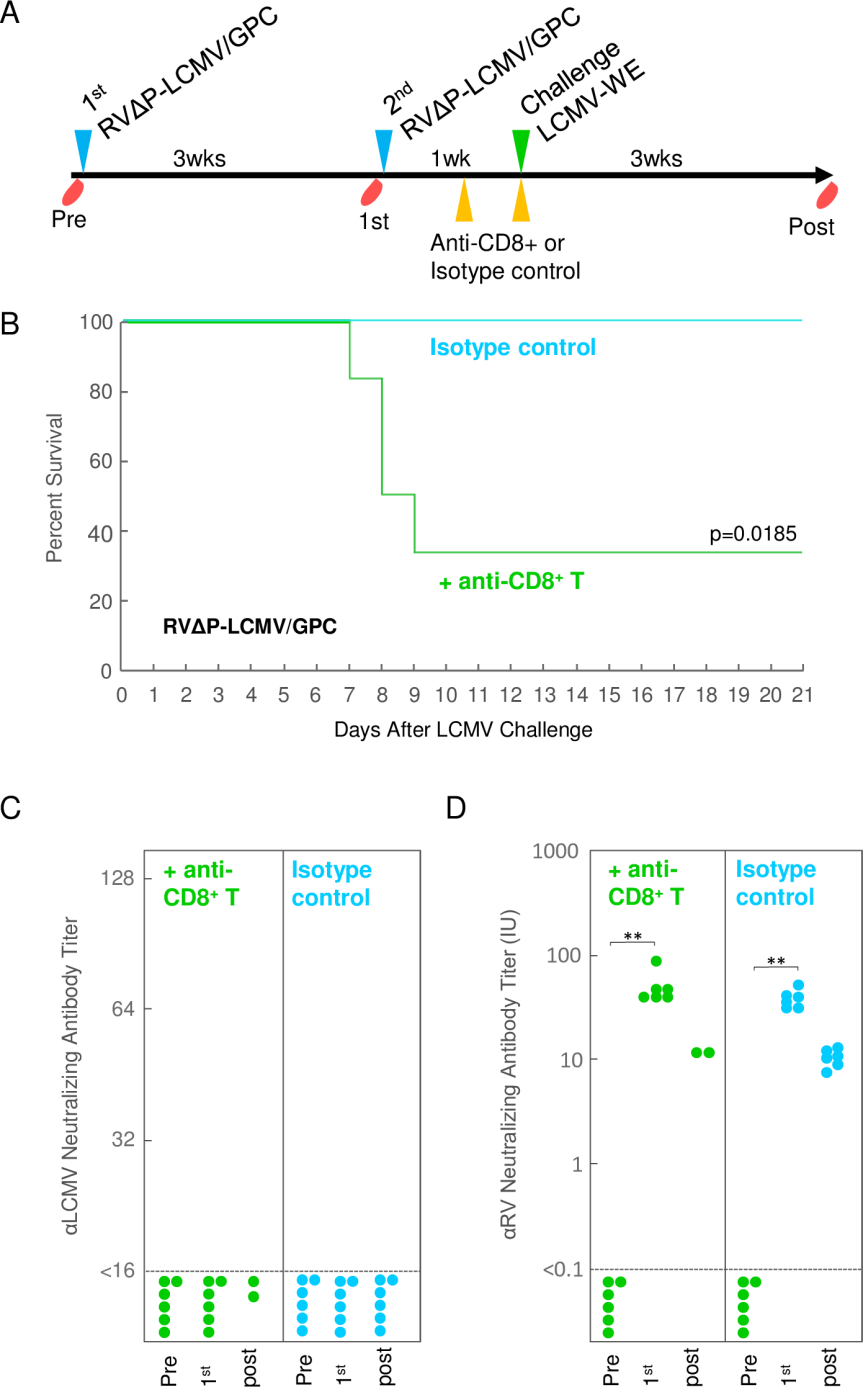
Survival curves and NAb titers in mice infected with LCMV-WE with or without CD8+ T cell depletion. (A) RVΔP-LCMV/GPC inoculation, challenge, and blood collection schedules in C57BL/6 mice. Mice were inoculated with RVΔP-LCMV/GPC twice at 3-week intervals. Mice were injected with anti-CD8+ T cell antibody (n = 6) or Isotype control (n = 6) 4 days and 7 days after the last RVΔP-LCMV/GPC inoculation. Seven days after the last inoculation, mice were intracranially infected with 10 PFU of LCMV-WE. Mouse serum was collected 1 day before the first RV inoculation (Pre) and second RV inoculation (1st), and the serum of the surviving mice was collected on the last day of the 3-week observation (Post). (B) The survival curve of mice injected with CD8+ T cell antibody or isotype control. The titers of anti-LCMV (C) and anti-RV (D) NAb in mice with and without CD8+ T-cell depletion.

To elucidate the involvement of CD8+ T cells in the protection against 10 PFU of LCMV infection in mice inoculated with RVΔP-LCMV/GPC, mice were inoculated with RVΔP-LCMV/GPC twice and injected with anti-CD8+ antibody before and after the LCMV challenge to deplete CD8+ T cells (Fig 6A). Alternatively, rat IgG2b immunoglobulin (isotype control) was administrated as a negative control. As shown in Fig 6B, depletion of CD8+ T cells apparently reduced to protect the mice inoculated with RVΔP-LCMV/GPC. The percent survival of CD8+ T-cell-depleted mice (33%) was significantly lower (p = 0.0185) than that of mice injected with isotype control (100%) (Fig 6B). The NAb titers against RV (Fig 6D) produced in sera of mice injected with anti-CD8+ antibody were the same as those in sera of mice injected with rat IgG2b immunoglobulin. This result indicated that the administration with these immunoglobulins did not affect the humoral immunity.

### Confirmation of the antigenic efficacy of the recombinant LCMV-GPC protein

**Fig 7:**
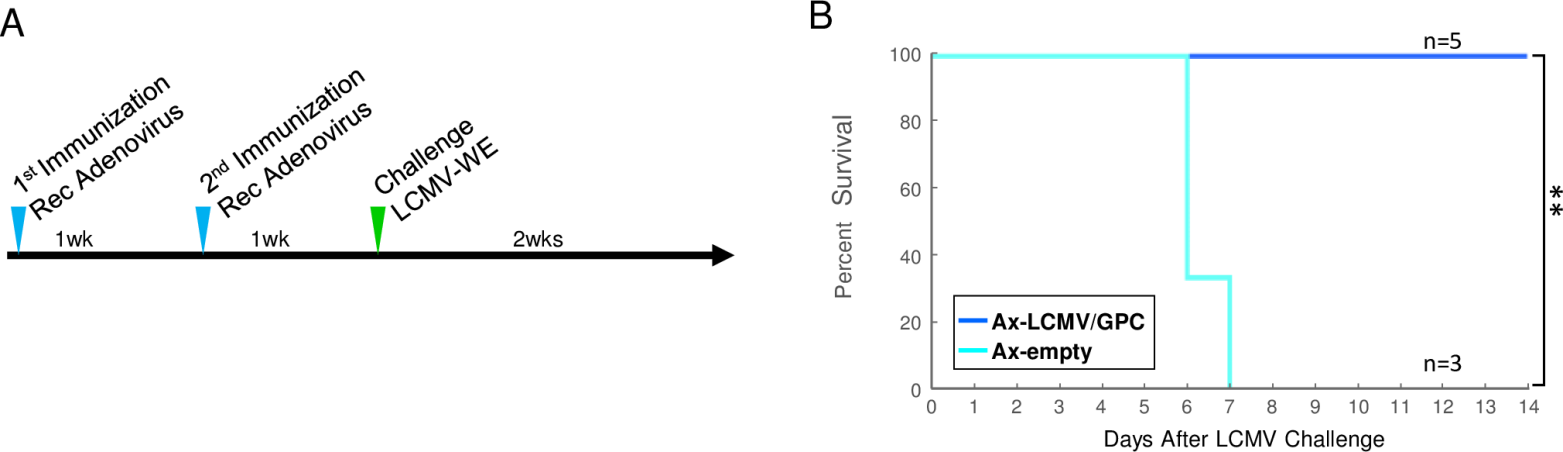
Survival curves in mice inoculated with adenovirus vectors before LCMV-WE infection. (A) The schedules of immunization with adenovirus and LCMV challenge in C57BL/6 mice. Mice were inoculated twice with Ax-LCMV/GPC or Ax-empty at 1-week intervals. One week after the last inoculation of adenovirus, mice were challenged with 10 PFU of LCMV-WE and observed for 2 weeks. (B) The survival curve of mice inoculated with Ax-LCMV/GPC or Ax-empty. Asterisks indicate a significant difference (p < 0.001).

Since the antigen expressed by viral vector is an important factor to elicit protective immunity against target virus. We confirm the antigenic efficacy of the expressed the LCMV-GPC which we used in this study, mice were immunized with Ax-LCMV/GPC and Ax-empty twice and challenged with 10 PFU of LCMV (Fig 7A). As shown in Fig 7B, all the mice inoculated with Ax-LCMV/GPC were survived after LCMV challenge, whereas all the mice inoculated with Ax-empty died (p = 0.0046). This result suggested that the LCMV-GPC sequences, which inserted in the genomes of RVΔP-LCMV/GPC and Ax-LCMV/GPC is sufficient for the antigen against LCMV protection.

## Discussion

The present study clearly demonstrated that RVΔP-LCMV/GPC, which cannot multiply but expresses LCMV and RV antigen, elicited protective immunity against LCMV and humoral immunity against RV in mice. Most of the mice inoculated with RVΔP-LCMV/GPC survived after LCMV challenge. RVΔP-LCMV/GPC simultaneously induced strong humoral immunity against RV. Moreover, we demonstrated that RVΔP-LCMV/GPC remained attenuated in suckling mice.

The main advantages of RV vectors are as follows: 1) RV can infect almost all mammals; 2) RV inoculation can elicit strong humoral and cellular immunity; 3) RV genomic RNA never integrates into the host genome because its replication cycle does not involve a DNA genome stage; and 4) globally, most people and animals have no immunity against RV. In addition, replication-incompetent RV is considered to be highly safe. Considering that the probable target population for receiving LCMV vaccine would include immunodeficient individuals, the safety profile of a candidate LCMV vaccine is extremely important. As we showed here, RVΔP-LCMV/GPC exhibited no pathogenicity in suckling mice inoculated intracerebrally. This suggests that RVΔP-LCMV/GPC would be avirulent in other mammals. Furthermore, it indicates that the presentation of endogenously synthesized viral antigens might stimulate responses of the cellular immunity in mice inoculated with RVΔP. Therefore, RVΔP-LCMV/GPC is considered to have the potential to elicit cellular immunity against LCMV. Although, the candidates of viral vector vaccines against LCMV have been reported such as adenovirus [54], vaccinia virus [55], influenza virus [56] and VSV [57], RV have some advantages mentioned above and have a long successful history as an inactivated vaccine for human and as a live vaccine for animals. Thus, RV could be a valuable alternative approach to the currently existing viral vectors.

Expression of the LCMV-GPC protein was clearly detected in BHK-P cells infected with RVΔP-LCMV/GPC. However, expression of the LCMV-GPC protein in infected Neuro-2a cells was not intensely detected by IFA, which indicated modest expression of the antigens even in cells without supply of the RV-P protein. In principle, RVΔP-LCMV/GPC did not multiply in cells not supplied with the RV-P protein, a constituent of the viral polymerase complex. The RV-P protein is incorporated into the RVΔP particle, so RVΔP could perform the primary transcription of viral mRNA even in cells without de novo synthesis of the RV-P protein [37]. Although the protein expression level *in vivo* was probably much lower than in the *in vitro* experiment, it would be sufficient to provoke an effective immune response. In fact, we showed that most of the mice immunized with RVΔP-LCMV/GPC survived the LCMV challenge and the sera from these mice had high neutralizing titers against rabies virus. Compared with adenovirus vector, the protection rate of RVΔP-LCMV/GPC was slightly lower than that of Ax-LCMV/GPC, this could be due to a difference of the expression level of the LCMV-GPC. We need further improvement to archive the higher expression level of the LCMV-GPC in the RVΔP RV vector system.

The slightly lower growth capacity of RVΔP-LCMV/GPC compared with that of RVΔP might be inversely correlated with the length of their RNA genomes. This modest decrease in viral growth has been observed in other studies [34,35]. Since the growth capacity of RVΔP-LCMV/GPC (highest titer 10^5^FFU/mL) is considerably lower than that of ordinary tissue-culture-adapted propagation-competent strains (e.g., 10^8^ FFU/mL), we are considering improving our preparation protocol or exploit other potential cell lines to obtain higher yields of virus stocks for vaccine production. Cells infected with RVΔP-LCMV/GPC or RVΔP showed no detectable cytopathic effects (CPE) and survived; thus, the culture could be maintained for nearly a month. This feature allows the repeated collection (up to three times) of culture fluid after sequential 6-day incubation periods. Unfortunately, it is expected that an extremely high dose of virus would be required for human use, so we should consider new strategies to overcome this limitation.

In this study, intraperitoneal inoculation twice with 10^6^ FFU of RVΔP-LCMV/GPC was shown to confer protective immunity against a challenge with LCMV. Anti-LCMV-GPC IgG antibodies were not detected before the LCMV challenge, demonstrating that neither NAb nor IgG antibodies were induced by the RVΔP-LCMV/GPC inoculation. Regarding LCMV infection, induction of NAbs against LCMV antigen is not important for protection and sometimes develops very late, namely, 60 to 120 days after infection [58]. It was reported that the administration of convalescent serum failed to prevent re-infection [1]. In fact, another study reported that Lassa-convalescent plasma did not significantly reduce mortality in any of the high-risk groups [59]. In contrast, Lassa-virus-specific cytotoxic T-cell responses were evident in patients who recovered from Lassa fever [60]. Anti-LCMV-GPC IgG antibodies were detected in the sera from surviving mice after the LCMV challenge, however these antibodies did not neutralize the LCMV. These results agree with the previous study in which mice exposed with high titer of LCMV developed GP-1-specific antibodies by day 8 but the antibodies failed to neutralize the virus [61]. As reported in a previous study [57], the LCMV-GPC itself, not the viral backbone, is responsible for the poor NAb response observed in mice infected with LCMV or recombinant VSV expressing the LCMV-GP. These findings suggest that cytotoxic T-cell responses are more important than the NAb titers for the protective immune response against arenaviruses. Accordingly, our results suggested that CD8^+^ T cells in mice inoculated with RVΔP-LCMV/GPC were essential to protect mice against a LCMV infection by the *in vivo* depletion of CD8^+^ T cells. As shown in Fig 6B, even though 33% of mice with CD8^+^ T cell depletion survived after LCMV challenge, the survival rate of those mice was significantly lower than that of the control mice (100%). Although, direct evidences of antigen-specific T cell responses should be monitored using ELISPOT assay or intracellular cytokine staining, it seems that CD8^+^ T cells play a critical role in the protective immunity against LCMV induced by RVΔP-LCMV/GPC inoculation. It is expected that RVΔP-LCMV/GPC would be a promising vaccine candidate for the practical development of a vaccine against LCMV.

Interestingly, inoculation with UV-irradiated RVΔP-LCMV/GPC exhibited a moderate efficacy for protection against LCMV challenge, it suggests that the inactivated preparation has provoked protective immunity against LCMV infection to some extent. Although, only a small amount of GP1 protein was detected in purified RVΔP-LCMV/GPC virus particles, it was clearly confirmed that its incorporation into these particles does occur. We suspect that this incorporated GP1 protein would stimulate immune responses in mice inoculated with UV-irradiated RVΔP-LCMV/GPC to some extent, albeit inefficiently, conferring partial protection against LCMV challenge. Inactivated vaccines are commonly believed to be weak inducers of cellular immunity. However, Papaneri et al. have reported that live and inactivated RV vaccines expressing Ebola virus glycoprotein (EBOV-GP) induced primary EBOV-GP specific T-cells and a robust recall response as measured by interferon-gamma ELISPOT assay [62]. This would be another unique feature of RV vectors. It is necessary to clarify how UV-irradiated RVΔP-LCMV/GPC could elicit any T-cell responses against the LCMV-GPC.

Protection against RV infection depends primarily on humoral immunity. A titer of NAbs greater than 0.5 IU/mL is indicative of fully positive seroconversion [63]. As expected, titers of anti-RV NAbs in mice inoculated with RVΔP-LCMV/GPC increased efficiently after the first and second inoculations. NAb titers remained above 0.5 IU during the post-challenge period. These results demonstrated that RVΔP-LCMV/GPC would induce high humoral immunity sufficient to protect mice against RV infection. Although RV is distributed worldwide, human mortality due to RV occurs mainly in Asia and Africa [64], which is somewhat similar to the mortality pattern observed for Old World arenaviruses, which is also concentrated in regions where they are endemic (West Africa and South America).

We have demonstrated that RVΔP-LCMV/GPC immunization successfully induced protective immunity against LCMV, probably associated with CD8^+^ T cells, concurrent with effective RV seroconversion. We are planning to precisely examine how RV vector elicits the T cell responses in future studies. We conclude that RVΔP-LCMV/GPC is a promising bivalent vaccine candidate against LCMV and RV. We expect that the RVΔP vector can be applied to develop highly attenuated vaccines against other arenaviruses, such as Lassa, Machupo, and Lujo viruses.
